# Cost effectiveness of recombinant factor VIIa for treatment of intracerebral hemorrhage

**DOI:** 10.1186/1471-2377-8-17

**Published:** 2008-05-19

**Authors:** Brett M Kissela, Mark H Eckman

**Affiliations:** 1 Department of Neurology, University of Cincinnati, 260 Stetson Street, Suite 2300, Cincinnati, Ohio 45267-05525, USA; 2 Division of General Internal Medicine and the Center for Clinical Effectiveness, University of Cincinnati, 231 Albert Sabin Way, Cincinnati, Ohio 45267-0535, USA

## Abstract

**Background:**

Phase I/II placebo-controlled clinical trials of recombinant Factor VIIa (rFVIIa) suggested that administration of rFVIIa within 4 hours after onset of intracerebral hemorrhage (ICH) is safe, limits ICH growth, and improves outcomes. We sought to determine the cost-effectiveness of rFVIIa for acute ICH treatment, using published Phase II data. We hypothesized that rFVIIa would have a low marginal cost-effectiveness ratio (mCER) given the poor neurologic outcomes after ICH with conventional management.

**Methods:**

We performed an incremental cost-effectiveness analysis from the societal perspective, considering conventional management vs. 80 ug/kg rFVIIa treatment for acute ICH cases meeting Phase II inclusion criteria. The time frame for the analysis was 1. 25 years: data from the Phase II trial was used for 90 day outcomes and rFVIIa complications – arterial thromboembolic events (ATE). We assumed no substantial cost differences in care between the two strategies except: 1) cost of rFVIIa (for an 80 mcg/kg dose in an 80 kg patient, assumed cost of $6,408); 2) cost of ATE side effects from rFVIIa (which also decrease quality of life and increase the chance of death); and 3) differential monetary costs of outcomes and their impact on quality of life, including disposition (home vs. nursing home), and outpatient vs. inpatient rehabilitation. Sensitivity analyses were performed to explore uncertainty in parameter estimates, impact of rFVIIa cost, direct cost of neurologic outcomes, probability of ATE, and outcomes after ATE.

**Results:**

In the "base case", treating ICH with rFVIIa dominates the usual care strategy by being more effective and less costly. rFVIIa maintained a mCER < $50,000/QALY over a wide range of sensitivity analyses. Sensitivity analyses showed that the cost of rFVIIa must exceed $14,500, or the frequency of ATE exceed 29%, for the mCER to exceed $50,000/QALY. Varying the cost and/or reducing the utility of health states following ATE did not impact results.

**Conclusion:**

Based on data from preliminary trials, treating selected ICH patients with rFVIIa results in lower cost and improved clinical outcomes. This potential cost-effectiveness must be considered in light of the Phase III trial results.

## Background

The majority of strokes worldwide are ischemic. Ischemic stroke victims that present within three hours are eligible for thrombolytic treatment with intravenous recombinant tissue plasminogen activator (r-tPA). This is a costly medication (retail price is approximately $2000) that is currently only given to a minority of patients [1,2]. Despite high costs and low utilization, the use of r-tPA is "cost-effective" at the population level over a 30 year time horizon [3].

While intracerebral hemorrhage (ICH) is much less common (approximately 10% of all strokes in the US),[4] it is a disease with high mortality and cost to society. Mortality at 30 days is 40–60% [4-6]A previous study in Cincinnati showed that many intracerebral clots expand in the short term; 38% of ICH's had > 33% expansion in first three hours and clot expansion was significantly associated with increased mortality and morbidity [7]. There are currently no medical therapies for ICH, and surgery to remove the blood clot is usually reserved only for severe, life-threatening ICH.

Recombinant factor VIIa (rFVIIa) is currently FDA-approved for the treatment of patients with hemophilia. Given its thrombogenic effects, it was hypothesized that it might be useful for limiting clot expansion in patients with acute ICH. Phase I and II placebo-controlled trials of rFVIIa have been completed in acute ICH patients [8,9]. These studies have demonstrated that administration of rFVIIa within the first 4 hours after onset of ICH symptoms is safe, limits ICH growth, and improves outcomes. Data from the Phase II trial (testing 3 doses) showed a dose effect, in that the 80 ug/kg dose was more effective than the 40 ug/kg dose. The 160 ug/kg dose results were not substantially better than for the 80 ug/kg dose, and the higher dose was associated with a slight increase in adverse events (serious thromboembolic events) [9]. ICH growth was 29% in the placebo arm as compared to 14% in the 80 mcg/kg group; mortality at 90 days was reduced from 29% in the placebo arm to 18% in the 80 mcg/kg rFVIIa arm. Safety was acceptable, but treatment was associated with a 2% risk of thromboembolic events.

Results from a phase III trial comparing the efficacy of 80 ug/kg rFVIIa vs. 20 ug/kg rFVIIa vs. placebo have been presented (by Stephan Mayer et al, at the 59^th ^Annual Meeting of the American Academy of Neurology, April 2007) but not yet published at the time of this writing. This trial did not demonstrate a clinical benefit for rFVIIa over placebo. However, a similar biologic effect was seen with regard to prevention of clot expansion.

Given the contradictory findings of the phase II and phase III trials, and the many issues raised about the phase III study, including questions regarding inclusion criteria and the surprisingly good outcomes in the placebo arm, there undoubtedly will be further discussion about the possibility of future trials.

In light of these issues, we wished to explore the cost-effectiveness of rFVIIa across a spectrum of neurological outcomes and efficacy of treatment, in order to better understand just how effective rFVIIa would have to be in order to be "cost-effective" for the acute treatment of ICH given the high cost and poor neurological outcomes in patients with acute ICH receiving conventional care. We studied this question by constructing a decision analytic model using published Phase II data for our base case analysis. Thus, we compared the neurological outcomes from the placebo arm (usual care; no treatment) of the Phase II trial to the results of treatment with rFVIIa at 80 ug/kg. We examined the impact of decreasing the efficacy of rFVIIa in preventing major neurological sequelae and death on the resultant cost-effectiveness of treatment.

## Methods

**Figure 1:**
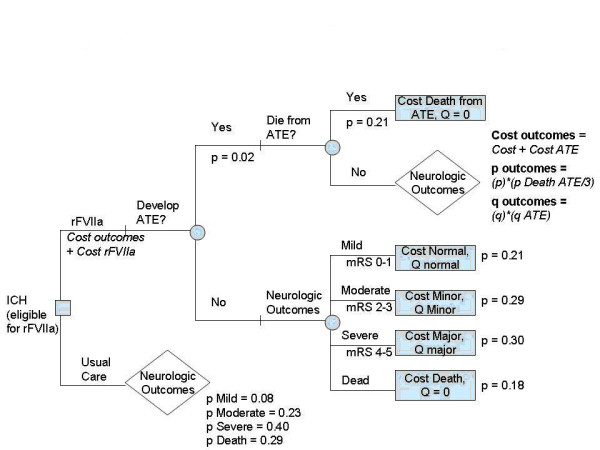


We used a standard computer program (Decision Maker)[10] to construct and analyze decision trees and to perform sensitivity analyses. Our analysis compares conventional supportive management (the current standard of care, as there are no other medical treatments for ICH) to treatment with rFVIIa (see Figure 1). We performed our cost-effectiveness analysis from a societal perspective, considering the decision only for those cases of ICH that would have met inclusion criteria in the Phase II trial. Previous estimates from a population- based study of ICH suggest that 13–17% of all ICH's would present within the 4 hour time window and be eligible; the most common exclusions from the Phase II trial were time to presentation and symptomatic thrombotic or vaso-occlusive disease within 30 days prior to ICH onset [11]. Sensitivity analyses were performed to explore uncertainty in parameter estimates, including the efficacy of rFVIIa in preventing major neurological sequelae and death. Further sensitivity analyses were performed to explore the impact of changes in the cost of rFVIIa, the direct costs of neurological outcomes, the probability of ATE, and outcomes after ATE. A marginal cost-effectiveness ratio (mCER) less than or equal to $50,000/QALY was considered to be below the threshold for societal 'willingness to pay', thus representing a reasonable expenditure of societal resources for the benefit gained.

## Decision Model Structure and Assumptions

### Model Structure and Parameters

We used the modified Rankin Scale (mRS) to describe neurological outcomes as in the Phase II trial. 9 Thus, patients receiving conventional management (lower branch of the decision node in Figure 1), may experience a range of neurological outcomes based on the distribution of 90 day mRS outcomes in the placebo arm in the Phase II trial.

Patients receiving treatment with rFVIIa (upper branch of the decision node) accrue the incremental cost of the drug. They next face the risk of arterial thromboembolism (ATE), the major adverse event associated with the use of rFVIIa. Patients who experience an ATE (either an ischemic stroke or myocardial infarction) may either die or survive. If they survive, they accrue additional costs for the care provided due to the ATE and also have a reduced quality of life due primarily to long-term neurological morbidity from ischemic stroke. If they do not have an ATE, they face a range of neurological outcomes, now modified by the efficacy of rFVIIa.

### Assumptions

Time Horizon of Analysis – We used a time frame for the analysis of one year. Data from the Phase II trial was used for 90 day outcomes, including incremental direct costs of care for use of rFVIIa and complications of ATE. We then modeled an additional 9 months to capture mid-term outcomes and costs, including direct costs accrued for nursing home (NH) stay if necessary, and for inpatient or outpatient rehabilitation in the post-ICH setting as appropriate to the mRS outcome. Since we hypothesized that rFVIIa would result in improved clinical outcomes and a mCER that would be acceptable from a societal perspective, any bias introduced into the analysis by not incorporating a longer analytic time horizon would be against the active intervention with rFVIIa. Therefore, if rVIIa is "cost-effective" in the shorter term, an analysis over the longer term would only accentuate the differences, by increasing the marginal effectiveness in long-term survivors with improved neurological outcomes, and possibly by decreasing the marginal cost.

### Cost

To simplify the analysis, only incremental direct costs during the index hospitalization were considered. We assumed that among patients eligible for rFVIIa, there would be no substantial cost differences in care between the two strategies (rFVIIa vs. usual care) except: 1) cost of rFVIIa; 2) cost of adverse events from rFVIIa; and 3) differential costs of outcomes, including disposition to home vs. NH and outpatient vs. inpatient rehabilitation. Given the short time frame of the model we did not discount costs.

The adverse event of greatest significance noted in the Phase II trial was the increased risk of arterial thromboembolism. We made the assumption that 50% of these ATE events were ischemic strokes (IS) and 50% were myocardial infarctions (MI) based on data from a recent review of complications with rFVIIa use [12]. Thus, to model the cost of the ATE complications, we used an average cost of inpatient stay for IS and MI (0. 5 × IS inpatient stay cost + 0. 5 × cost of MI inpatient stay) as provided in the 2006 American Heart Association Statistical Update [13].

We made several simplifying assumptions about the costs of the various neurologic outcomes. For mRS 0–1 ("mild impairment"), we assigned outpatient rehabilitation costs only. Patients with a mRS of 2 can usually go home with assistance after ICH, while those with a mRS of 3 usually require NH residence. Thus half of the mRS 2–3 patients ("moderate impairment") were assigned NH costs and half were discharged to home with outpatient rehabilitation costs (no incremental NH costs). For mRS 4–5 ("severe impairment"), we assigned permanent NH residence for one year.

## Review of the Data

### Clinical Outcomes and Utilities

We used previously published utilities for stroke outcomes derived from time-trade off analyses. These studies have shown that time-trade off data are least prone to biases [14]. In our model, a mRS of 4–5 mapped to an ICH with "severe" neurological sequelae, while mRS of 2–3 and 0–1 mapped to "moderate" and "mild" sequelae, respectively.

Utilities for mRS 0–1 were derived from Gage et al [15]. Utilities for mRS 2–3 and 4–5 are derived from Post's systematic review of the literature [14]. For each outcome state, a base case estimate and a utility range are presented, including a negative utility for mRS of 4–5 since some patients consider this outcome to be worse than death (see Table 1). Ranges are modeled upon those used by Fagan et al [3]. Survivors of ATE were arbitrarily assigned a 55% decrease in the expected utility for the post-ICH mRS (from the 80 ug/kg group) due to the further insult of the ATE. This adjustment in utility is based upon the assumption that the major impact of ATE's would be ischemic strokes with "moderate" long-term neurologic impairment.

**Table 1:** Model parameters

	**Probabilities**				
**Variable**	***No RX***	***rFVIIa (40 mcg)***	***rFVIIa (80 mcg)***	**Utilities**	***Range***

Mild post-ICH deficit (mRS 0–1)	0. 08	0. 17	0. 21	0. 81	0. 70–0. 95
Moderate post-ICH deficit (mRS 2–3)	0. 23	0. 29	0. 29	0. 55	0. 2–0. 65
Severe post-ICH deficit (mRS 4–5)	0. 40	0. 37	0. 30	0. 25	(-0. 2)-0. 45
Death after ICH	0. 29	0. 18	0. 18	0	0
Arterial thromboembolic event		0. 02	0. 02	0. 55	
Probability of dying after ATE		0. 29	0. 29		

### Probabilities and Rates

The rate of ATE among all patients treated with rFVIIa in the Phase II trial was 2% [9]. The assigned probability of dying after an ATE was 29%, based on data from a recent review of complications with rFVIIa use [12].

### Costs

Information about the cost of rFVIIa was obtained from the University of Cincinnati pharmacy. For an 80 mcg/kg dose in an 80 kg patient, the cost of rFVIIa is $6,408 (personal communication, K. Sangha, PharmD, 2/16/2006). Costs of rehabilitation and NH residence were derived from Fagan et al and Holloway et al;[3,16] 1998 costs were adjusted to 2005 US dollars via The Inflation Converter [17]. Costs (and ranges for sensitivity analyses) are presented in Table 2.

**Table 2:** Economic assumptions

**Outcomes**	Costs*	**Range**
NH residence (one year)	$46,346	$23,175 – $57,939
Inpatient rehabilitation	$24,720	$11,588 – $46,351
Outpatient rehabilitation	$2,591	$1,391 – $2,897
Severe stroke(mRS 4–5)	$46,346	$23,175 – $57,939
Moderate stroke (mRS 2–3)	$35,533	$15,000 – $45,000
Mild stroke (mRS 0–1)	$2,591	$1,391 – $2,897
rFVIIa	$6,408	
Arterial thromboembolic event (ATE)	$9,261	

* 2005 US dollars.

## Results

In the "base case" analysis, the strategy of treating ICH with rFVIIa dominates the usual care strategy by being both more effective and less costly (see Table 3).

**Table 3:** Base case analysis

Strategy	Cost ($)	Effectiveness (QALYs)	Marginal Cost ($)	Marginal Effectiveness (QALYs)	Marg C/E ratio (Δ$/ΔQALYs)
rFVIIa	$39,305	0. 40			
Usual Care	$40,359	0. 29	$1,053. 20	-0. 11	********

******** no mCER because rFVIIa dominates usual care by being both more effective and less costly.

We performed a variety of one-way sensitivity analyses to determine how robust our results were in the face of some uncertain input parameter values. We also wanted to explore potential future changes in the cost of therapy and the evolving efficacy data for rFVIIa. First, we varied the direct cost of neurological outcomes to the highest and lowest costs in the proposed range, respectively. Not surprisingly, when costs of poor neurologic outcome are higher than the base case, treatment with rFVIIa dominates conventional therapy. At the lowest costs for outcomes in our range, costs become virtually identical; rFVIIa is more expensive (by only $14) but has significantly greater effectiveness, favoring the rFVIIa strategy with a mCER of $129 per QALY.

**Figure 2:**
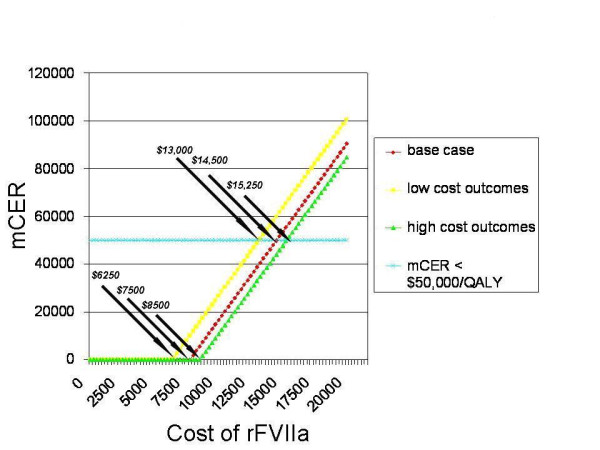


We also performed sensitivity analyses on the cost of rFVIIa. The base case cost per dose of rFVIIa is $6,408. rFVIIa remains the dominant strategy unless the cost per dose exceeds $7,500. At higher costs per dose, the mCER remains less than $50,000/QALY until the cost per dose surpasses $14,500. These results are presented in Figure 2, along with sensitivity analyses in which the lowest and highest costs of neurologic outcomes were varied from the base case costs. Even if we assume the lowest cost of neurologic outcomes in the model, the rFVIIa strategy is 'cost effective' (less than $50,000/QALY) until the cost per dose exceeds $13,000, and dominates unless the cost per dose exceeds $6,250, which is just slightly lower than the base case cost of $6,408.

**Figure 3:**
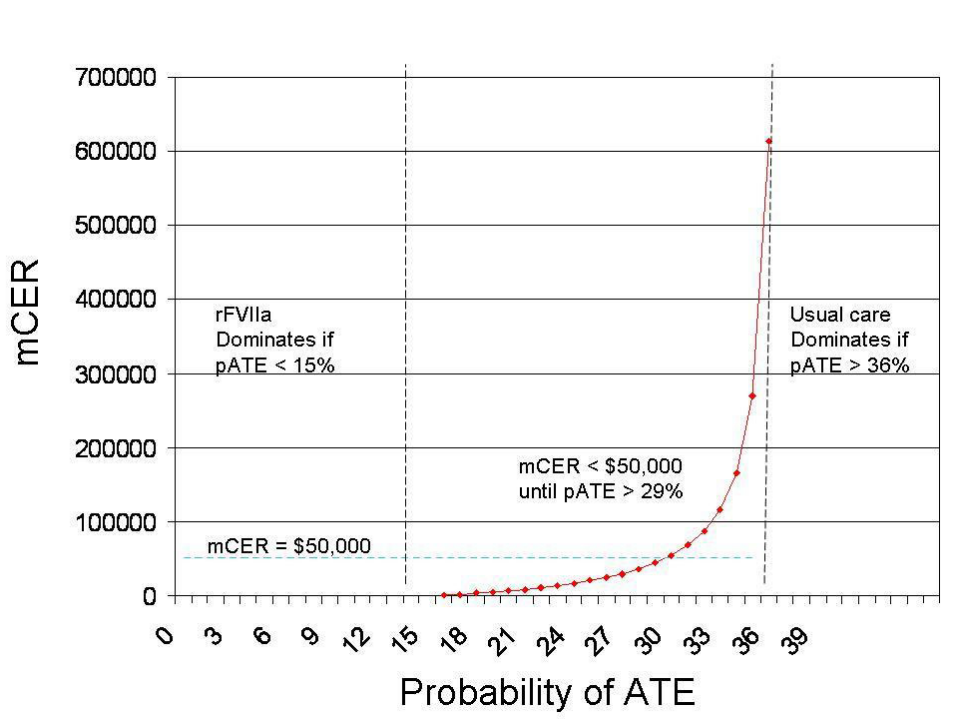


In sensitivity analyses examining the probability of arterial thromboembolic complications (ATE), rFVIIa dominates conventional treatment unless the probability of ATE exceeds 15% (base case – 2%) and the mCER remains less than $50,000/QALY unless the probability of ATE exceeds 29%. Conventional therapy becomes less costly and more effective only if the probability of ATE exceeds 36%. Results are presented in Figure 3.

Since efficacy is frequently overestimated in early clinical trials, we also explored the impact of changes in the efficacy of rFVIIa to ensure that our results would not underestimate the mCER of rFVIIa. We first considered the possibility that the true efficacy might be only as good as the results for the 40 ug/kg dose from the Phase II trial (see Table 1). Even when substituting these less effective neurological outcomes into the base case model, treatment with rFVIIa costs little for the benefit it produces with a mCER of $9,541 per QALY.

Further multi-way sensitivity analyses were performed assuming the lower efficacy of the 40 mcg/kg dose. When using these outcomes and varying the probability of developing ATE, the ATE rate must still exceed 18% for the cost-effectiveness ratio to exceed $50,000/QALY. In another analysis using the lower efficacy of the 40 ug/kg dose and varying the cost of rFVIIa, the rFVIIa strategy dominates until the drug cost exceeds $5,250 and the mCER does not exceed $50,000/QALY until the drug cost is greater than $10,750.

**Figure 4:**
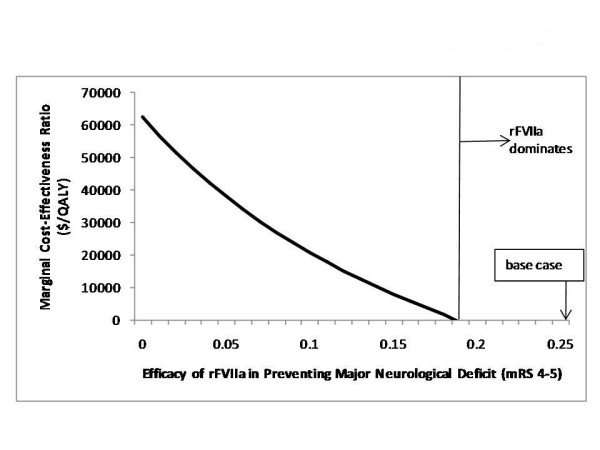


**Figure 5:**
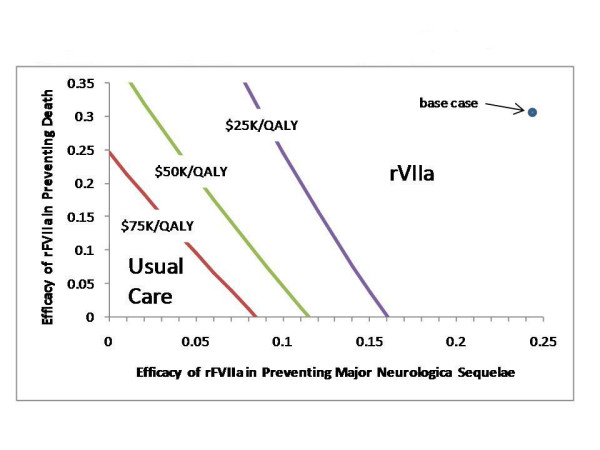


We next explored the impact of changes in the efficacy of rFVIIa in preventing major neurological sequelae (i. e., mRS 4–5) and in the efficacy of rFVIIa in preventing death. As shown in Figure 4, as the efficacy in preventing major neurological impairment increases, the marginal cost-effectiveness of rFVIIa becomes smaller. Beyond an efficacy of 18%, rFVIIa is both less expensive and more effective than usual care. In Figure 5, we explore both the efficacy of preventing major neurological sequelae (on the horizontal axis) and the efficacy in preventing death (on the vertical axis). A series of lines is shown for 3 different thresholds for willingness-to-pay, $25K/QALY, $50K/QALY, and $75K/QALY. These thresholds separate the analytic space into two regions, one in which rVIIa is best (the upper right) and one in which usual care is best (the lower left). The base case values for these two efficacy terms from the phase II study fall to the upper right, in the region in which rFVIIa is favored. If one believes the results of the phase III trial are correct (efficacy in preventing major neurological sequelae and in preventing death equal zero), then usual care is best. For any combination of efficacies greater than those falling along the $50K/QALY willingness-to-pay threshold, rFVIIa has a reasonable cost for the benefit gained. Should future studies demonstrate these efficacies to be greater than those falling along this threshold line, rFVIIa would be "cost-effective."

The results were insensitive to changes in a number of other parameters including the probability of dying after an ATE and the utility for neurological outcomes in survivors of ischemic stroke or myocardial infarction (due to ATE).

## Discussion

Based upon the published results of the phase II trial, the treatment of eligible ICH patients with rFVIIa would be promising from both a clinical and economic point of view, resulting in better outcomes at a lower cost than conventional management. Our model is simple, and only examines short-term cost-effectiveness, but accounts for most of the relevant marginal costs and our conclusions did not change over a variety of sensitivity analysis. The bias introduced into our analysis by a short time horizon should only make rFVIIa appear more costly and less effective. Extending the time horizon of the analysis would only increase the marginal effectiveness and perhaps decrease the marginal cost of rFVIIa.

A more sophisticated modeling approach involving a Markov state transition model has recently been published [18]. The findings of this manuscript were similar to ours in that rFVIIa was highly cost-effective. This analysis examined the full range of doses tested in the Phase II study, and while all doses were cost-effective the 80 ug dose was both more effective and less costly. Their model was robust, with stability across a wide range of 1-way sensitivity analyses. Their results have the advantage of Markov modeling across a longer time span, and took into account long-term disability, expected mortality, and cost multipliers (with appropriate discounting over time). Their findings demonstrate that the high cost of poor outcomes have much greater impact than medication costs, and that extending the model to longer time frames only enhances the incremental costs associated with poor outcome (in the sensitivity analyses, the annual cost multiplier had the greatest impact on the incremental cost-effectiveness ratio). This confirms that short term analyses such as ours only bias against rFVIIa, and make our findings of cost-effectiveness in the short term more robust.

In this recent publication, arterial thromboembolic events that could result from treatment with rFVIIa were not considered. It is important to consider such adverse events, as they impact upon costs, mortality, and long-term outcome for survivors. Furthermore, patients with vaso-occlusive disease were excluded from the Phase II trial, and in an analysis of eligibility across a population, vaso-occlusive disease was one of the most common exclusions (for 36% of ICH patients) and thus significantly impacts upon applicability of this therapy. It should be noted that the Phase III trial did not exclude patients with a history of vaso-occlusive disease, presumably to increase generalizability. Our data suggest that doing so is reasonable up to a point, as treatment with rFVIIa remains 'cost-effective' at higher levels of ATE.

Earnshaw et al [18]. found that drug costs only accounted for between 1. 5–6% of the total lifetime medical costs in their model. We found that the cost of rFVIIa would need to be increased substantially before the treatment strategy exceeds the traditional threshold societal willingness to pay of $50,000/QALY.

Our model is limited by the short time horizon considered, and the consideration of direct incremental costs only. Earnshaw et al [18] included indirect medical costs such as caregiver burden in their model. Inclusion of such costs would only be expected to decrease the marginal cost-effectiveness of treatment with rFVIIa.

At this time, there is no alternative medical treatment for ICH. The benefits seen in the Phase II trial were promising, and even though only a minority of ICH patients would be eligible for treatment with rFVIIa with the Phase II trial inclusion and exclusion criteria, our results indicate that such treatment is highly cost-effective and within society's willingness to pay.

Results of the Phase III study (r**F**VIIa in **A**cute Hemorrhagic **S**troke **T**reatment; FAST trial) have recently been presented (Mayer et al, AAN meeting). These as yet unpublished results showed that the rFVIIa intervention did not result in superior clinical outcomes at 3 months, which was the primary clinical endpoint of the trial. If these results are reproducible and generalizable, the use of rFVIIa would not be cost-effective as its use would result in added cost with no increase in effectiveness. Perhaps, the real answer lies somewhere in between the results of these two studies. The sensitivity analysis shown in Figure 5 allows us to consider the impact of future trial results that fall somewhere along this spectrum.

The discrepancy seen between the early clinical trials and the FAST trial was discussed in the FAST results presentation. The data presented suggest a similar biologic effect as noted in the Phase II trial, in that rFVIIa significantly limited hematoma expansion. However, based on a pre-planned interim analysis, the sample size was increased due to fewer poor outcomes than anticipated. In presenting the FAST trial results, the investigators noted that the placebo group in the FAST trial had much better 3-month outcomes than the placebo group in the Phase II trial, leading to discrepant results despite a similar biologic effect. In post-hoc analyses exploring the results, between-group heterogeneity was seen for several key factors related to mortality and outcome, especially for the oldest patients in the trial, despite adequate randomization. For example, the rFVIIa treated arms (20 ug and 80 ug) had a higher proportion of patients with cardiac disease (left ventricular hypertrophy pattern on EKG) and a higher proportion of patients with intraventricular extension of the hematoma. Notably, all three arms had similar rates of venous thromboembolic events, and the high dose rFVIIa arm had a 10% rate of ATE vs. 5% in the placebo arm.

A post-hoc analysis performed by the investigators, suggested that the benefit of rFVIIa might have been realized in the Phase III trial had there been minor changes in inclusion and exclusion criteria, Specifically, the post-hoc analysis suggested that benefit would be greatest if one excluded patients with later treatment (> 180 minutes) and age > 75.

## Conclusion

In light of our results, and those of Earnshaw and colleagues, we believe that another Phase III trial should be considered. Our analysis shows that the ATE rates seen in the FAST trial are well within the limits of potential cost-effectiveness, and given that the biologic effect was similar in the Phase II trial and the FAST trial similar cost-effectiveness might be found with appropriate revision of the inclusion/exclusion criteria. We await the publication of the FAST results so that our model can be applied in the hopes of providing estimates of this potential cost-effectiveness. Until further studies of this type are performed, ICH remains a disease with high morbidity and mortality, with no effective medical treatment.

## Competing interests

The authors declare that they have no competing interests.

## Non-financial competing interests

Dr. Kissela was a Site Co-Investigator in the Phase III rFVIIa trial, sponsored by Novo Nordisk. Dr. Eckman is an editorial consultant for the American College of Physicians – Physicians' Information and Education Resource (PIER) module on pre-operative assessment for bleeding disorders.

## Authors' contributions

BK conceived of the study and performed the data review. Together, BK and ME developed the model parameters, designed the model, analyzed the model results, and generated the figures for the manuscript. BK drafted the manuscript. Both authors read and approved the final manuscript.

## Pre-publication history

The pre-publication history for this paper can be accessed here:


